# 17beta-estradiol (E2) Regulates Malignancies and Stemness in Endometrial Carcinoma (EC) via Interacting with ESR1

**DOI:** 10.1007/s43032-025-01871-1

**Published:** 2025-05-08

**Authors:** Xiaochao Xu, Xinzhi Dai, Cheng Huang, Xia Guan, Cuiwei Zhang

**Affiliations:** 1 College of Food and Biological Engineering, Chengdu University, Chengdu, 610000 Sichuan China; 2 The First Clinical lnstitute, Zunyi Medical University, Zunyi, 563000 Guizhou China; 3 Department of Pathology, The Affiliated Hospital of Southwest Medical University, Luzhou, 646000 Sichuan China; 4 Precision Pathology Diagnosis for Serious Diseases Key Laboratory of LuZhou, Luzhou, 646000 Sichuan China; 5 Department of Pathology, Sichuan Provincial Maternity and Child Health Care Hospital, Chengdu, 610000 Sichu P.R. China

**Keywords:** Endometrial carcinoma (EC), Epithelial-mesenchymal transition (EMT), Estrogen receptor-1 (ESR1), Endometrial cancer stem-like cells (ECSCs), 17beta-estradiol (E2)

## Abstract

Endometrial cancer is one of the most common and fatal gynaecologic malignancies and is associated with the presence of estrogen. Endometrial cancer stem-like cells (ECSCs) are stem-like cell subpopulations endowed with self-renewal and differentiation capacities and are critical for EC progression. However, it is still unknown whether estrogen is involved in regulating stemness in EC and contributes to malignancies. Therefore, we investigated the regulatory effects of E2 treatment on oestrogen receptor-1 (ESR1).

ESR1 expression was measured in EC tissues; After ESR1 overexpression of knockdown, the effects of E2 treatment was evaluated, including cell proliferation, sphere formation, invasion and colony formation. Our results indicate that ESR1 is critical for the regulatory effect of E2 on EC cells. After being cultured in serum-free medium, the ECSCs were found to be enriched in stemness markers, including CD133, CD44 and ALDH1. Interestingly, enriched ECSCs presented significantly lower expression levels of these factors than the parental cells. Overexpression of ESR1 slightly affected stemness and malignant potential in ECSCs, and the addition of 2 nM E2 markedly decreased stemness and inhibited malignant behaviours of ECSCs, including proliferation, invasion and tumour formation, in soft agar. Moreover, the addition of E2 significantly inhibited the expression of epithelial-mesenchymal transition (EMT) markers, including ZEB1, ZEB2, and E-cadherin.

ESR1 is critical for regulating EC progression in the presence of E2, partially by regulating stemness in ECSCs, indicating that ESR1 might be a potential therapeutic target for EC.

**Supplementary Information:**

The online version contains supplementary material available at 10.1007/s43032-025-01871-1.

## Introduction

According to novel EC classification, it can be broadly classified into four biologically distinct EECs: POLE-ultramutated EC, mismatch repair-deficient EC, p53-mutant EC and no specific molecular profile EC [1, 2]. Clinically, the majority (approximately 85%) of endometrial cancer cases are type I endometrial cancers, which are oestrogen-dependent endometrioid adenocarcinomas. Oestrogens exert their biological activity by binding to oestrogen receptors, primarily ERα, which regulate the expression of multiple genes associated with the development and progression of endometrial cancer [3–6]. Type I endometrial tumours, also known as low-grade adenomas exhibit low-grade glandular structures. These cells typically express high levels of oestrogen receptor α (ERα) and are considered hormone-driven [7, 8]. Type II tumours include high-grade adenomas, serous tumours, clear cell tumours, carcinosarcomas, and tumours with mixed histology. These tumours express ER to a lesser extent [9, 10], have a poorer prognosis, and share molecular features similar to those of triple-negative breast cancer and serous ovarian cancer, including a high frequency of p53 mutations and copy number alterations [11]. Although type II endometrial cancer has a poorer prognosis, the incidence of hormone-driven type I endometrial cancer is higher, and it accounts fo more deaths [12].

Estrogens exert their biological activity by binding to ERα and ERβ, mediating cellular responses to hormone exposure. Compared to ERα, ERβ is typically expressed at very low levels in the adult uterus [13]. Moreover, it is widely recognized that the action of oestrogens is mediated primarily by ERα in endometrial cancer. Oestrogens play a role in promoting mitosis in the normal endometrium as part of the tissue growth associated with the expected pregnancy during the menstrual cycle. In the late follicular phase of the menstrual cycle, oestrogens (particularly estrone and 17β-oestradiol, or E1 and E2) are produced by developing follicles, leading to endometrial growth [9]. In animal models, high levels of oestrogen without counteraction by progesterone lead to endometrial hyperplasia or carcinogenesis [12–15], indicating that a lack of oestrogen/progesterone balance may contribute to the early development of endometrial cancer.

In a recent review of risk factors and the incidence of endometrial cancer, body mass index (BMI) was strongly associated with cancer risk in both premenopausal (relative risk per 5 kg/m2 increase = 1.49) and postmenopausal women (relative risk per 5 kg/m2 increase = 1.60) [16]. The link between oestrogen and obesity stems from the ability of adipose tissue to synthesize oestrogens [18]. At reduced levels, progesterone is unable to significantly inhibit the oestrogen-stimulated growth of the endometrium [17], resulting in uncontrolled growth of endometrial cells. Patients with polycystic ovary syndrome (PCOS) have an increased risk of developing endometrial cancer [18], and part of this risk may be related to anovulation. In addition to hereditary cancer syndromes such as Lynch syndrome [19] and Cowden syndrome [20], oestrogen signalling is one of the key driving forces for the development of endometrial cancer.

Cancer stem cells (CSCs) are a population of tumour cells that share similar characteristics with normal stem cells, including self-renewal, multipotent differentiation, and unlimited proliferation. CSCs were initially discovered in acute myeloid leukaemia and have since been confirmed in various solid tumours [21, 22]. Recently, the presence of CSCs has been considered a process of evolution in cancer progression [23]. CSCs are closely associated with chemotherapy resistance, endocrine therapy resistance, cancer recurrence, invasion, and metastasis [24–26]. Emerging research has also indicated the presence of CSCs in endometrial cancer tissue, as these cells can form clones and initiate tumour formation [27]. Although the existence of endometrial stem cells has been widely recognized, whether tumour stem cells are influenced by oestrogens has not been reported, and these findings may provide valuable information for the clinical treatment of endometrial cancer.

In this study, we enriched tumour stem cells from endometrial cancer cells and observed the relationship between the expression of ESR1 and the malignant behaviour of tumour stem cells induced by oestrogens. We explored the impact and potential underlying mechanisms of ESR1 on the malignant behaviour of tumour stem cells under oestrogen stimulation and provided insights for clinical treatment.

## Materials and Methods

### Samples and Ethics

This study was approved by the Clinical Experiment Ethics Committee of Southwest Medical University Affiliated Hospital (NO.KY2024206, Date of Approval: May 22, 2024). Human tumor samples were obtained on June 1, 2024. The tumor samples were obtained from 15 patients diagnosed with endometrial cancer in the tissue sample library. During and after data collection, all identifying information was anonymized and securely stored. Only authorized personnel could access this information, following the institution’s data protection and ethical guidelines, and only aggregated data was used for analysis.

### H&E Staining and Immunohistochemistry

He matoxylin (HHS16, Sigma-Aldrich) and eosin (HT110232, Sigma Aldrich) staining of samples were performed according to the manufacturer’s instructions. The samples were used for immunostaining of ESR1(ab108398, ABcam, UK), which was performed following the streptavidin-biotin alkaline phosphatase complex method using the Vectastain ABC-AP standard kit (Vector Laboratories; Burlingame, CA).

### GEPIA Analysis

The correlation analysis between ESR1 expression and survival outcomes was performed using the GEPIA2 online platform (http://gepia2.cancer-pku.cn) with TCGA pan-cancer cohort data. Specifically, TCGA-STAD RNA-seq expression data (FPKM normalized) and corresponding clinical survival information were retrieved through the GEPIA2 interface. For survival analysis, patients were stratified into “high-expression” and “low-expression” groups based on the median ESR1 mRNA level as the cutoff threshold. Kaplan-Meier survival curves were generated using the log-rank test with a significance threshold of *p* < 0.05. Hazard ratios (HR) with 95% confidence intervals were calculated through Cox proportional hazards regression. The analysis considered overall survival (OS) as the primary endpoint.

### Western Blotting

Proteins were collected from paraffin-embedded tissues and cultured cells, and then blotted by the antibodies mentioned below. We used SDS-PAGE to separate proteins, transferred them to PVDF membranes, and examined them with the relevant primary antibodies specific for ESR1(ab108398), CD24 (ab179821), CD133 (ab278053), CD44 (ab189524), β-actin(ab8227) was used as a loading control. All these antibodies were bought from Abcam. All experiments were repeated for three times.

### Cell Culture and Treatment

Ishikawa and RL95-2 cells, two types of human endometrial carcinoma cell lines, were acquired from American Type Culture Collection (ATCC Manassas, VA, USA). These cells were cultured in Dulbecco Modified Eagle Medium (DMEM, Gibco, Paisley, UK), supplemented with 10% fetal bovine serum (FBS, Gibco, Paisley, UK), as well as 100 U/ml pencillin and 100 U/ml streptomycin at a 37℃ incubator with 5% CO2. To isolate endometrial cancer stem cells (ECSCs) from Ishikawa and RL95-2 cells, the cells were maintained in DMEM/F12 without FBS. The culture medium was supplemented with 2% B27 supplement (Life Technologies, Grand Island, NY, USA), 20 ng/ml human EGF (PeproTech, Rocky Hill, NJ, USA), 40 ng/ml bFGF (PeproTech, Rocky Hill, NJ, USA), and 5ug/ml insulin (PeproTech, Rocky Hill, NJ, USA). Every three days, medium was half-refreshed. The cells were regularly passaged every 10 to 14 days.

### Plasmid Constructions and Generation of Stable Cells

Previously, the construction of the estrogen-receptor α (ERα) eukaryotic expression vector was described as follows. Briefly, an approximately 1788 base pairs (bp) coding sequence was PCR-amplified from MCF-7 total RNA, which was reverse-transcribed into cDNA. The amplification was performed using specific primers: P1,5’-GCCGGCGCTAGCATGCCATGACCCTCCACACCA-3’ (enzyme site: Nhe I) and P2, 5’-CCTTAACTTAAGCAGACCGTGGCAGGGAAACCC-3’ (enzyme site: Hind III). The amplified fragment’s accuracy was confirmed by DNA sequencing. It was then double digested with Nhe I and Hind III and subsequently cloned into the pcDNA3.1 vector (Life Technology) containing the neomycin resistance gene. For introducing shRNA targeting to ESR1, pLV3-U6-ESR1(human)-shRNA1-CopGFP-Puro and empty vector were bought from KEMOBio (China, Zhejiang). For transfection, the Lipofectamine TM 2000 transfection reagent (Life Technology) was used according to the manufacturer’s instructions. To establish stable transfectants, cells were transfected with either the pcDNA3.1-ERα vector or the pcDNA3.1 vector. After 24 h of.

transfection, 400 μg/ml G418 was added and maintained for a period of 4 weeks.

### Propidium Iodide (PI) Staining Followed by Flow Cytometry

Cells were trypsinized and resuspended in ice-cold PBS. After centrifugation, the cells were fixed with ice-cold 70% ethanol. After a 4-hour incubation period, the cells were pelleted again and washed with ice-cold PBS. Subsequently, the cells were suspended in a solution containing 5 μg/ml propidium iodide (PI, Sigma-Aldrich, St. Louis, MO, USA) and incubated for 10 min in darkness. Finally, the stained cells were analyzed using three laser Navios flow cytometers (Beckman Coulter, Brea, CA, USA) by following the manufacture’s instruction. All experiments were repeated for three times.

### Cell Proliferation

A total of 1 × 10 3 cells were plated in 24-well plates and allowed to incubate overnight. Following that, the cells were treated with 2 nM of E2 or ethanol (mock) for 24 h. The volume of ethanol added to the mock wells was equivalent to that added to the wells treated with the drug. Subsequently, the medium was removed, and the wells were washed three times with PBS. The plates were then frozen at -20℃ overnight for further processing using the CCK-8 Proliferation Assay Kit(Life Technology). The absorbance at OD450-620 was measured and recorded. All experiments were repeated for three times.

### Tumor Formation in Soft Agar

To evaluate the ability of tumor formation in vitro, soft agar clonogenic assays were performed. In each well of a 6-well plate, 2 mL of 0.5% (w/v) low-melting agar (Sigma–Aldrich, St. Louis, MO, USA) in DMEM medium with 10% FBS was prepared. The cells were mixed evenly, and then 5 × 10 3 cells in 2 mL of 0.3% low-melting agar in 10% FBS were added on top of the solidified base solution. The plates were incubated at 37℃ with 5% CO_2_ for a period of 14 days, allowing the colonies to form. Afterward, colonies were imaged under a microscope. Then, in each randomized view, the number and diameter of the colonies were quantified by using Image J software (version-2.0; U.S.-National Institutes of Health; Bethesda-Maryland, USA). All experiments were repeated for three times.

### Transwell Invasion Assay

The cells were treated with 0.25% Trypsin, followed by three washes with ice-cold PBS. Then, 2 × 10 ^5^ cells were added to the top chamber of inserts, which contained polycarbonate filters with 8 μM pores (Corning Incorporated, Corning, NY, USA). These filters were pre-coated with a Matrigel membrane (BD Biosciences, Franklin Lakes, NJ, USA). The experiments were conducted in triplicate. After a 24-hour incubation period, the cells on the upper membrane were removed, and the invaded cells were stained with crystal violet (Beyotime Institute of Biotechnology, Beijing, China). Finally, the invaded cells were counted under a microscope at a magnification of ×100. All experiments were repeated for three times.

### Statistical Analyses

Data are presented as the mean ± standard error of the mean. All statistical analyses were performed using SPSS 26.0 software (SPSS, Inc., Chicago, IL, USA). The differences between two groups were compared using a paired Student’s ttest and the differences between three or more groups using oneway analysis of variance followed by Tukey’s posthoc test. Probabilities less than 5% (*P* < 0.05) were regarded as statistically significant. All experiments were repeated three or more times to ensure reliability.

## Results

### In EC, the Expression Level of ESR1 Increases and Is Positively Correlated with Overall Survival

**Fig. 1:**
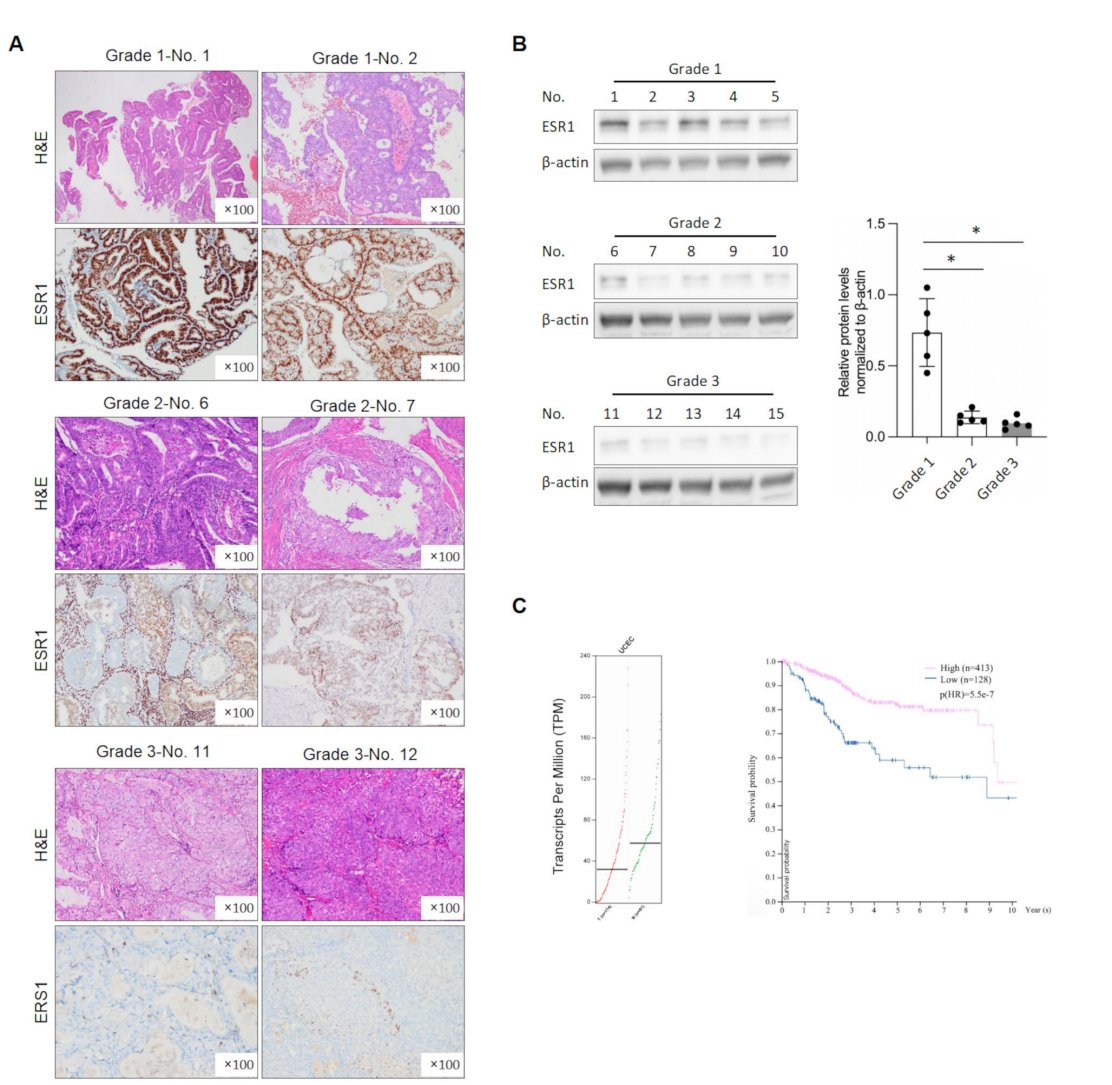
ESR1 expresses differently depending on EC progression and positively correlated with total survival time. **A**. 5 samples, for Grade 1, Grade 2 or Grade 3 of EC were employed to detect ESR1 expression by immune-histochemistry. **B**. Total protein of these EC samples were collected and expression of ESR1 was detected by performing western blot. **C**. By comparing with the TCGA database, it was employed to detect the transcriptional level of ESR1 in EC tissues compared to adjacent tissues, and then, the correlation of ESR1 expression with total survival time was compared. *p < 0.05 vs. Grade 1

We collected tumor samples from patients with Grade 1, 2, and 3 endometrial cancer using FlGO grading criteri, determined the pathological features through H&E staining. Immunohistochemistry was used to detect the protein expression levels of ESR1. The results showed that the protein level of ESR1 in Grade 1 endometrial cancer tumor samples was significantly higher compared to Grade 2 and 3 samples (Fig. 1A), which was consistent with the trend of ESR1 protein content in sample lysates detected by western blot, indicating higher levels of ESR1 protein in Grade 1 endometrial tumor samples (Fig. 1B). Furthermore, via the analysis of TCGA-STAD database by an online tool GEPIA, we found a positive correlation between high expression of ESR1 and survival rate in endometrial cancer (Fig. 1C).

### The Expression of ESR1 in EC Cells Has Little Impact on the Malignant Behavior of the Cells Without E2 Treatment

To further confirm the role of ESR1 in endometrial cancer, we selected the endometrial cancer cell lines Ishikawa (ISK, ESR1 positive) and RL95-2 (ESR1 weak) for further investigation (Supplementary Fig. 1). We stably overexpressed ESR1 in RL95-2 cells by transfection with an overexpression vector, and efficiently knocked down ESR1 in ISK cells by transfection with a shRNA vector.

**Fig. 2:**
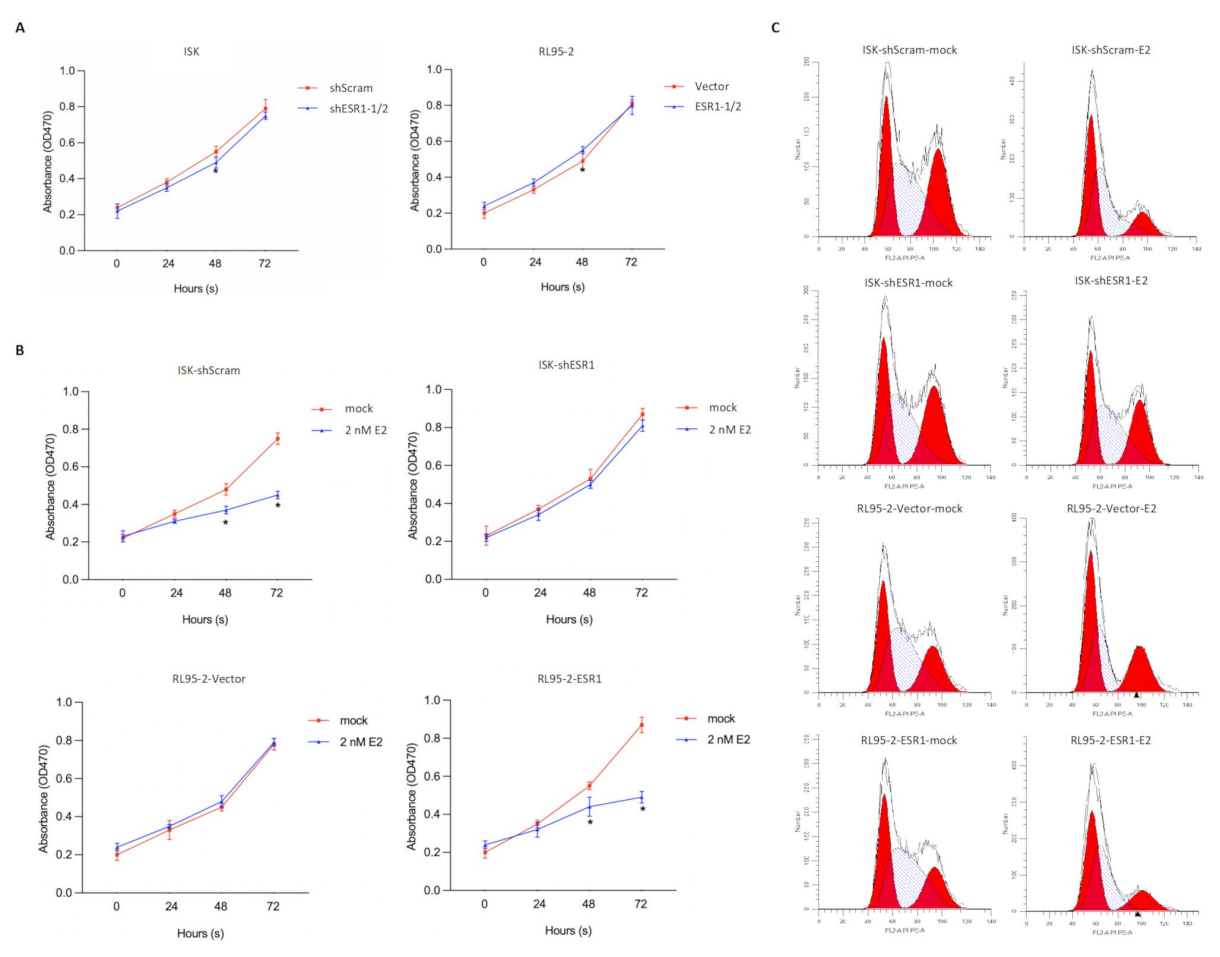
Treatment of E2 affects proliferation in EC cells via the presence of ESR1. (**A**) After efficiently knockdown of ESR1 in ISK or overexpression of ESR1 in RL95-2 cells, CCK-8 assay was performed from 0 to 72 h to detect the effect of ESR1 expression on cell viability. With the addition of 2 nM E2, CCK-8 assay was performed at 0–72 h to detect cell viability (**B**) and cells were respectively collected, stained by PI and analyzed by flow cytometry to detect cell phase distribution (**C**)

After regulating the expression level of ESR1, the cell viability was not affected during the first 1–5 days (Fig. 2A). However, when treated with 2 nM E2, the proliferation rate of ISK-shScram significantly decreased, while ISK-shESR1-1/2, which is a mixture of two clones stably expression shRNA targeting to ESR1, showed no significant response to the added E2. The cell viability of RL95-2-ESR1 significantly decreased after E2 treatment, while RL95-2-vector was not affected by E2 (Fig. 2B). We further evaluate the effect of E2 on cell cycle phase distribution with the presence of ESR1. Expectedly, with the presence of ESR1, E2 blocked cell cycle phase at G1/G0, indicating that in EC cells, 2 nM of E2 blocked cell cycle phases (Fig. 2C).

### ESR1 Has an Inhibitory Effect on the Enrichment of Cancer Stem Cells Derived from EC Cells, and this Effect Is Dependent on E2

**Fig. 3:**
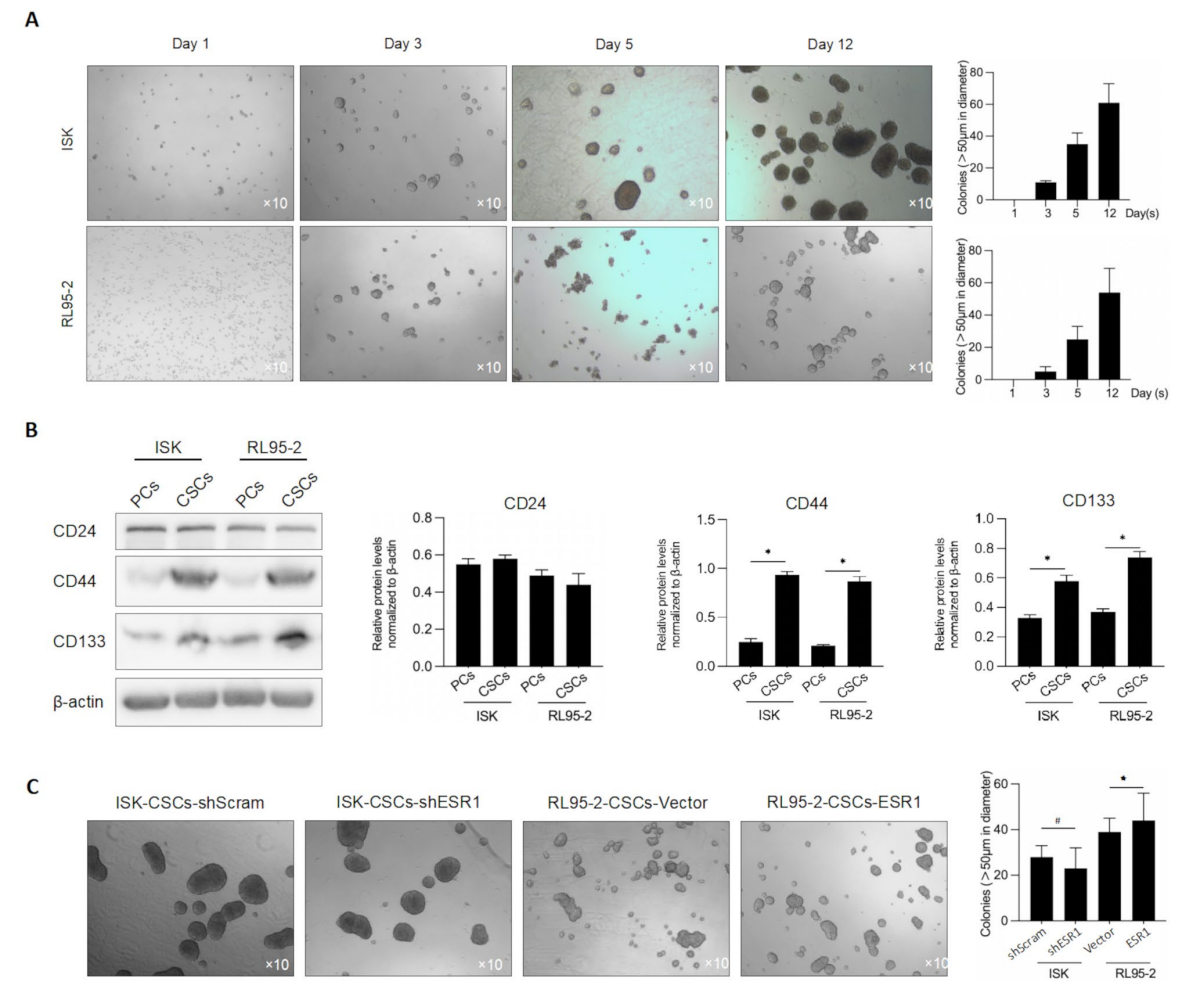
Collection and identification of cancer stem-like cells (CSCs) in ECs. **A**. After being cultured in serum-free medium with addition of EGF, bFGF and B27 for 1, 3, 5 and 12 days, formed spheres were imaged. **B**. The stemness hallmarkers, including CD24, CD44, CD133 were detected by western blot. *p < 0.05 vs. PCs group. **C**. After efficiently knockdown of ESR1 in ISK or overexpression of ESR1 in RL95-2 cells, sphere formation was detected after being cultured for 12 days. ^#^*p* < 0.05 vs. ISK-CSCs-shScram, *p < 0.05 vs. RL95-2-CSCs-Vector

**Fig. 4:**
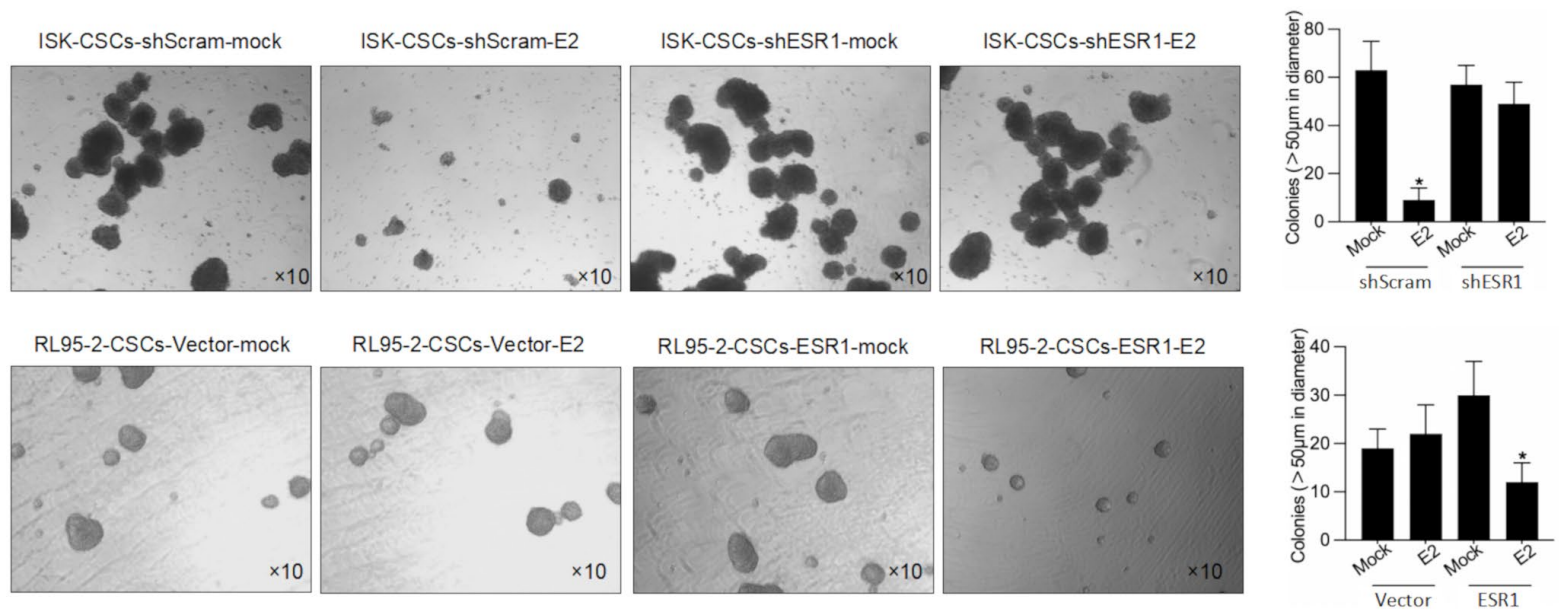
Presence of E2 inhibits sphere formation in EC. After efficiently knockdown of ESR1 in ISK or overexpression of ESR1 in RL95-2 cells, with the presence of 2nM E2 or not, sphere formation was observed after 12 day-culture in serum-free medium. *p < 0.05 vs. mock group

After 12 days of cultivation in serum-free medium, it was observed that distinct cell spheroids formed in all cell lines (Fig. 3A), accompanied by a significant increase in the expression levels of stemness-related factors (Fig. 3B). This suggests that under serum-free conditions, cancer stem cells were able to proliferate and form cell spheroids, exhibiting characteristics of stemness. Through serial-replating assay experiments, it was found that the expression level of ESR1 in the third generation had no apparent impact on the self-renewal ability of cancer stem cells (Fig. 3C). This indicates that the expression level of ESR1 does not directly influence the proliferation and renewal capacity of cancer stem cells. However, when 2 nM of E2 was added to the culture of the stem cell spheroids and continued for 72 h, it was observed that the stem cell spheroids of ISK-shScram and RL95-2-ESR1 disintegrated, while ISK-ESR1 and RL95-vector were unaffected (Fig. 4). This suggests that E2 has an inhibitory effect on the maintenance of stemness in stem cells, and this effect is dependent on the presence of ESR1. Taken together, ESR1 inhibits the enrichment of cancer stem cells derived from EC cells by regulating the effects of E2. Further research has revealed that ESR1 has an inhibitory effect on the enrichment of cancer stem cells derived from EC cells, and this effect is dependent on the presence of E2. ESR1 plays a crucial role in endometrial regulation through the mediation of hormone pathways. Given that the expression level of ESR1 is negatively correlated with the clinical stages of endometrial cancer, it is speculated that ESR1 may be involved in the occurrence or differentiation process of cancer stem cells.

### The Expression of ESR1, in the Presence of E2, Inhibits the Malignant Behavior of Stem Cells

**Fig. 5:**
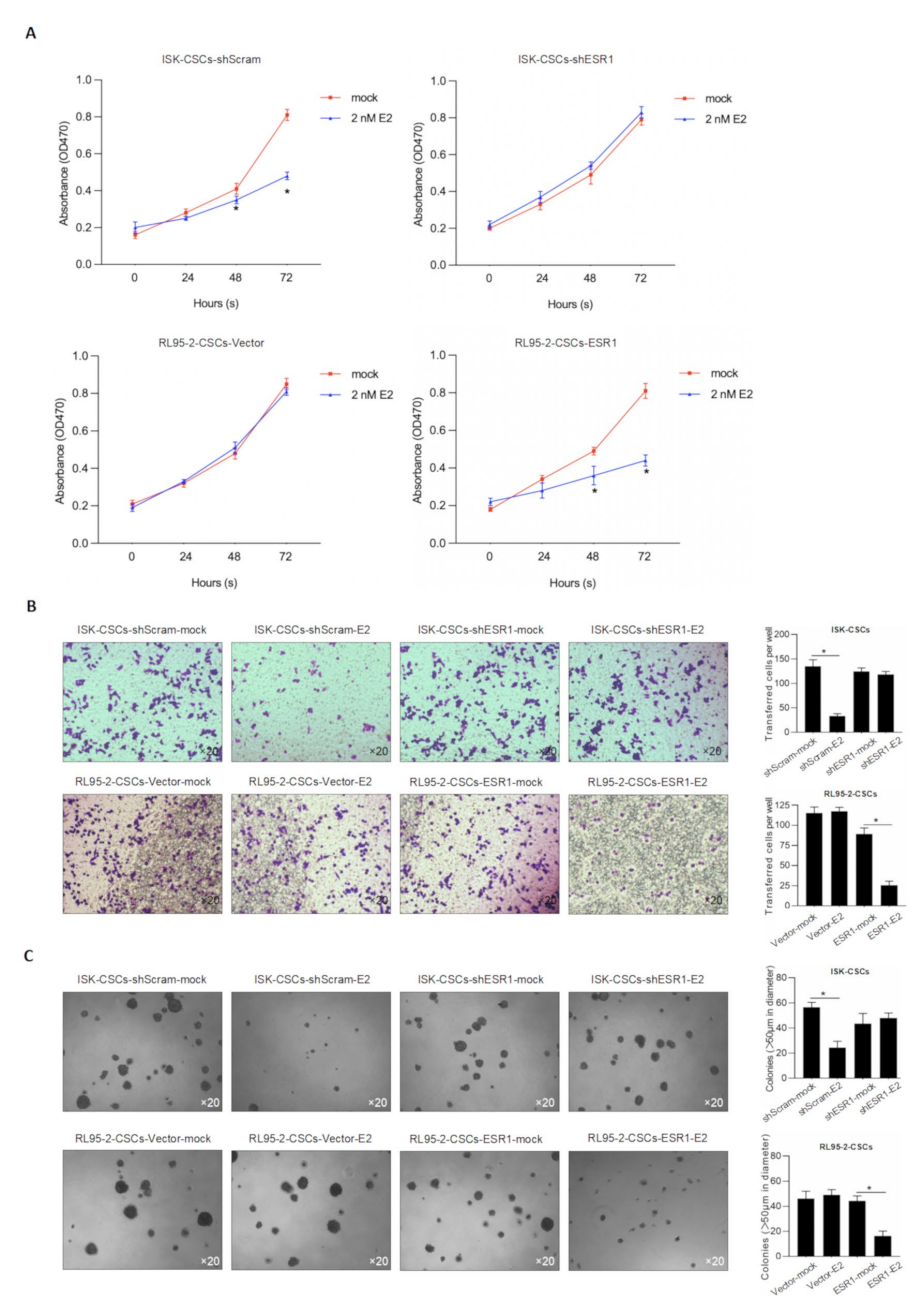
E2 treatment reduced malignant behaviors in EC CSCs via interacting with ESR1. **A**. After efficiently knockdown of ESR1 in ISK CSCs or overexpression of ESR1 in RL95-2 CSCs, the effects of 2 nM E2 on cell viability from 0 to 72 h were detected by performing CCK-8 assay. ^*^P < 0.05 vs. mock group. **B**. transwell assay was employed to detect the effect of E2 on cell invasion. **C**. tumor formation in soft agar was performed to detect the effect of E2 on tumor formation

Previous results indicated that E2 has an impact on the stemness of stem cells. Therefore, does the loss of stemness caused by E2 result in changes in the malignant behavior of stem cells? To confirm this question, we added E2 to the groups with or without interference of ESR1 and then assessed the cell proliferation activity, invasive ability, and soft agar colony formation ability for 1–5 days. As shown in Fig. 5A, treatment with E2 led to a significant decrease in cell proliferation ability. However, after interfering with ESR1, the inhibitory effect of E2 on cell proliferation became less evident.

**Fig. 6:**
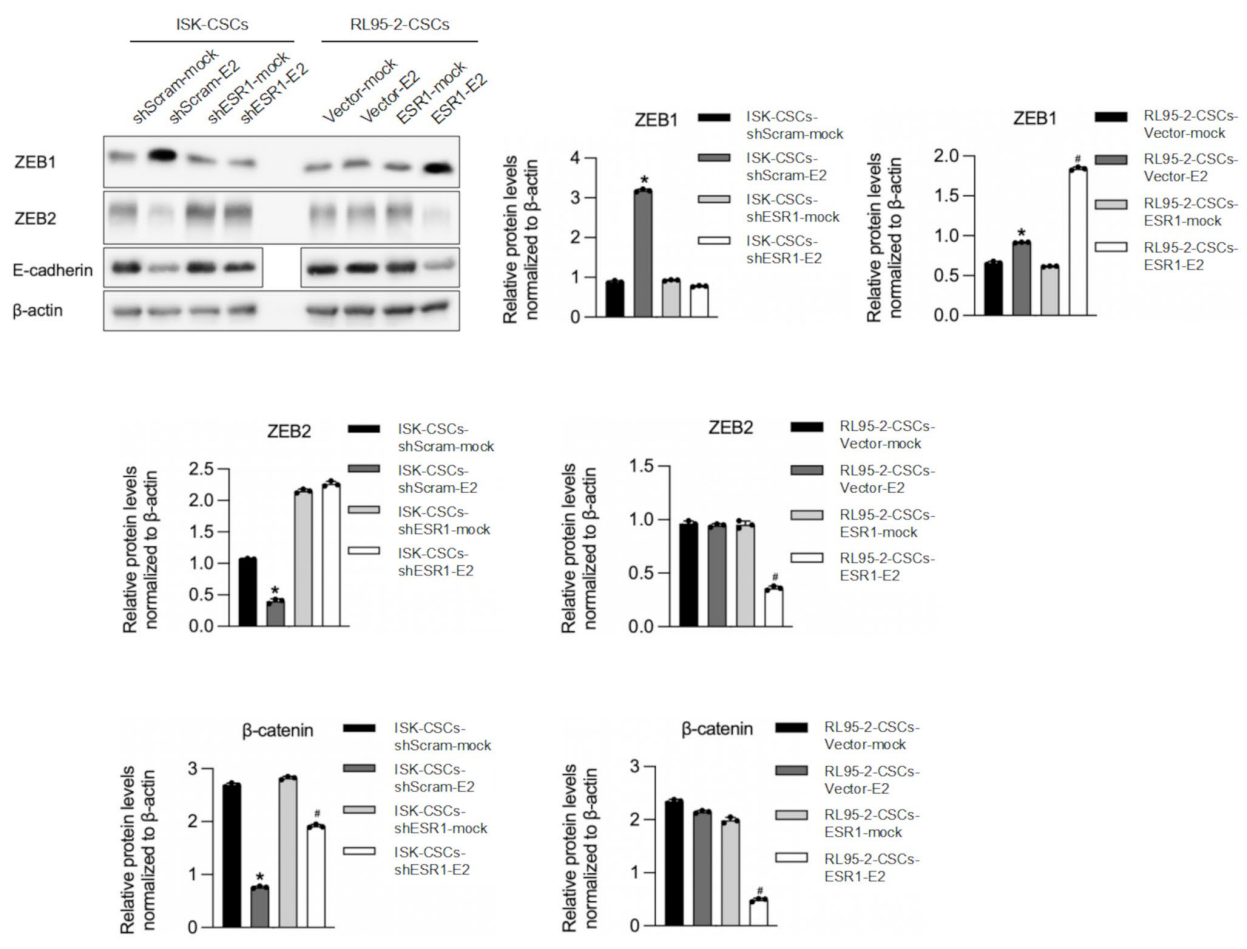
E2 treatment reduced EMT via interacting with ESR1. After efficiently knockdown of ESR1 in ISK CSCs or overexpression of ESR1 in RL95-2 CSCs, the effects of 2 nM E2 on hallmarker genes of EMT, including ZEB1, ZEB2 and E-cadherin were detected by performing western blot. *p < 0.05 vs. ISK-CSCs-shScram-mock, #*p* < 0.05 vs. ISK-CSCs-shESR1-mock

Consistently, we found that the addition of E2 resulted in a decrease in cell invasive ability, but after interfering with ESR1, the inhibitory effect of E2 on invasive ability became less evident (Fig. 5B). We also examined the effect of E2 on the tumorigenic ability of cancer stem cells. The results demonstrated (Fig. 5C) that the addition of E2 suppressed the tumor formation ability of cancer stem cells, and after interfering with ESR1, the inhibitory effect of E2 became less evident. Further, we also examined the expression of hallmark genes of epithelial-mesenchymal transition (EMT), including ZEB1, ZEB2, and E-cadherin (Fig. 6). The results showed that the addition of E2 inhibited the process of EMT, and this effect was dependent on the presence of ESR1.

## Discussion

We analyzed estrogen receptor alpha (ESR1) expression in endometrial cancer (EC) tissues and adjacent noncancerous tissues. Contrary to expectations, ESR1 transcriptional levels were significantly lower in tumor tissues, yet paradoxically, high ESR1 expression correlated with improved patient survival. Further investigation revealed that tumor stem cells (TSCs) isolated from EC tissues lost stemness and exhibited suppressed malignancy upon E2 treatment. This observation aligns with the survival advantage in high ESR1-expressing patients, as TSCs—key drivers of metastasis and recurrence—were effectively inhibited. Our findings suggest that E2 suppresses TSC stemness via ESR1, thereby improving prognosis in EC.

EC progression is hormone-dependent. Estrogen-induced histone acetylation is well-documented in EC pathogenesis [28–29], and prolonged estrogen exposure elevates acetylation levels in mammary tissues [30]. Here, we demonstrated that E2 interacts with estrogen receptors to modulate EC malignancy, consistent with prior studies [12, 29]. While ESR1 expression alone did not directly affect TSC stemness or malignancy, E2 treatment markedly suppressed these properties. Mechanistically, E2-activated ESR1 inhibits TSC-driven processes such as chemoresistance, metastasis, and recurrence. These results underscore ESR1 as a critical regulator of TSC activity and EC prognosis.

Although estrogen is central to EC development, other genes (e.g., PTEN, K-Ras, hMLH1, p53) also contribute [31–34]. Our data confirm that E2 exerts minimal effects on ESR1-deficient EC cells but significantly suppresses malignancy in ESR1-overexpressing cells. Notably, E2 attenuated epithelial-mesenchymal transition (EMT) in TSCs in an ESR1-dependent manner. While EMT’s role in TSC maintenance remains unclear, our work establishes ESR1 as a key mediator of E2’s anti-TSC effects. Further studies are needed to resolve whether E2 inhibits stemness via EMT suppression.

Type 1 EC evolves under sustained estrogen signaling, mediated genomically/non-genomically by ERα/ERβ and GPR30 [35–36]. While prior studies implicate LSD1 in estrogen-regulated pathways [37], we focused on ERα/β in TSCs, leaving GPR30’s role unexplored. A key limitation is our use of two EC cell lines, insufficient to capture EC’s genomic heterogeneity. Additionally, in vitro findings require validation in vivo to assess E2/ESR1 effects on tumor progression. Future work should incorporate diverse EC subtypes and patient-derived models.

Therapeutically, ESR1-targeting agents like Fulvestrant (a SERD effective in breast cancer) may inhibit ESR1-driven TSC signaling in EC. Combining ESR1 antagonists with EMT modulators (e.g., ZEB1/ZEB2 inhibitors) could enhance efficacy. In conclusion, while our study identifies ESR1 as a regulator of TSC biology in EC, translational applications demand in vivo validation, broader model systems, and preclinical testing of ESR1-directed therapies.

## Electronic Supplementary Material

Below is the link to the electronic supplementary material.

Supplementary Material 1

## Data Availability

All relevant data are within the manuscript and its supporting information files.
