# Supplementary material for: 17beta-estradiol (E2) Regulates Malignancies and Stemness in Endometrial Carcinoma (EC) via Interacting with ESR1
Source: Reprod Sci. 2025 May 8;32(7):2295–306. doi: 10.1007/s43032-025-01871-1 (PMC12271265; doi:10.1007/s43032-025-01871-1)
Supplement: Supplementary file 1 — Supplementary Material 1 [file 43032_2025_1871_MOESM1_ESM.docx]

**
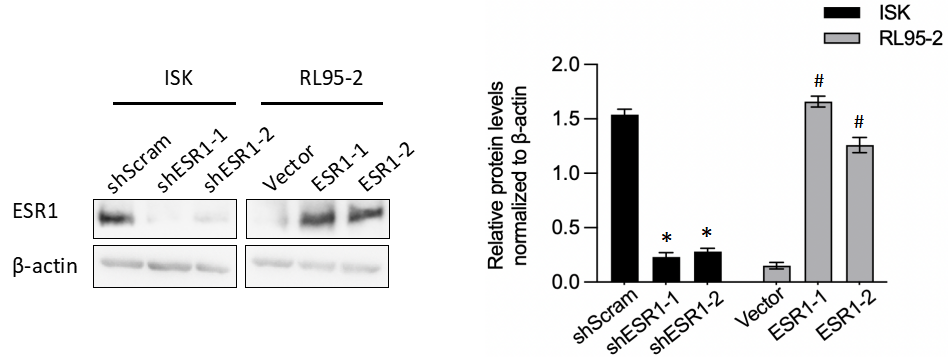
**

**Supplementary figure 1. ESR1 was efficiently modified in EC cells**

In ISK cells, ESR1 was efficiently knockdown by transfecting shRNA targeting to ESR1 mRNA. **p* <0.05 *vs* ISK-shScram, In RL95-2 cells, ESR1 was efficiently overexpressed by transfecting plasmid with coding sequence of ESR1. #*p* < 0.05 *vs* RL95-2-Vector.
